# Lung Cancers Treated With Photodynamic Therapy and Surgery

**DOI:** 10.1155/DTE.5.155

**Published:** 1999

**Authors:** Tetsuya Okunaka, Toshimitsu Hiyoshi, Kinya Furukawa, Hideki Yamamoto, Takaaki Tsuchida, Jitsuo Usuda, Hideo Kumasaka, Junzou Ishida, Chimori Konaka, Harubumi Kato

**Affiliations:** Department of Surgery Tokyo Medical University 6-7-1, Nishihinjuku Shinjuku-ku Tokyo 160-0023 Japan

## Abstract

Laser endoscopic surgery, especially the effectiveness of photodynamic therapy (PDT) using Photofrin as a photosensitizer, has now achieved a status as effective treatment modality for lung cancer. Twenty-six lung cancer patients received the preoperative PDT for the purpose of either reducing the extent of resection or increasing operability. Bronchoscopical PDT is performed with topical anesthesia approximately 48 h after the intravenous injection of 2.0 mg/kg body weight of Photofrin. Operation was performed 2–9 weeks after initial PDT. The initial purpose of PDT, i.e. either to reduce the extent of resection or convert inoperable disease to operable status, was achieved in 22 out of 26 patients treated. The survival rate of T3 (main bronchus invasion) cases treated by surgery alone increased significantly from 50.9% to 60.0% with the application of preoperative PDT. This remarkable result may imply that this new option of PDT as preoperative laser irradiation may contribute to the management of advanced lung malignancy.


*Diagnostic and Therapeutic Endoscopy*, Vol. 5, pp. 155–160
Reprints available directly from the publisher
Photocopying permitted by license only

© 1999 OPA (Overseas Publishers Association) N.V.
Published by license under
the Harwood Academic Publishers imprint,
part of The Gordon and Breach Publishing Group.
Printed in Malaysia.

### Lung Cancers Treated with Photodynamic Therapy and Surgery

TETSUYA OKUNAKA*, TOSHIMITSU HIYOSHI, KINYA FURUKAWA, HIDEKI YAMAMOTO, TAKAAKI TSUCHIDA, JITSUO USUDA, HIDEO KUMASAKA, JUNZOU ISHIDA, CHIMORI KONAKA and HARUBUMI KATO

*Department of Surgery, Tokyo Medical University, 6-7-1, Nishihinjuku, Shinjuku-ku, Tokyo 160-0023, Japan*

**Laser endoscopic surgery, especially the effectiveness of photodynamic therapy (PDT) using Photofrin as a photosensitizer, has now achieved a status as effective treatment modality for lung cancer. Twenty-six lung cancer patients received the preoperative PDT for the purpose of either reducing the extent of resection or increasing operability. Bronchoscopical PDT is performed with topical anesthesia approximately 48 h after the intravenous injection of 2.0 mg/kg body weight of Photofrin. Operation was performed 2–9 weeks after initial PDT. The initial purpose of PDT, i.e. either to reduce the extent of resection or convert inoperable disease to operable status, was achieved in 22 out of 26 patients treated. The survival rate of T3 (main bronchus invasion) cases treated by surgery alone increased significantly from 50.9% to 60.0% with the application of preoperative PDT. This remarkable result may imply that this new option of PDT as preoperative laser irradiation may contribute to the management of advanced lung malignancy.**

*Keywords:* Combination therapy, Lung cancer, Photodynamic therapy

### INTRODUCTION

Laser endoscopic surgery has now achieved a status as effective treatment modality for lung cancer. Especially increasing attention has been focused on photodynamic therapy (PDT) using Photofrin. Over the past decade, 248 patients (296 lesions) with central type lung cancers have been treated in our hospital. Overall complete remission (CR) was obtained in 42.5% of the 125 lesions, partial remission (PR) in 56.8% and no remission was obtained in 1.0% [1]. Indications of PDT are as follows: (1) Early stage lung cancer as a curative purpose: among 126 early stage lesions CR was obtained in 107 (84.9%) and 61 cases were disease free at 2–200 months [2]. (2) Advanced lesions for opening of bronchi: overall, "effective" opening of bronchi was achieved in 61 out of 81 lesions (75%) for the PDT group, as opposed to 143 of 177 (81%) for the Nd-YAG laser therapy group [3].

* Corresponding author. Tel.: 813-3342-6111, ext. 5071. Fax: 813-3349-0326.

(3) Preoperative laser irradiation for the purpose of increasing operability and reducing the extent of operation: the initial purpose of PDT, i.e., either reduction of extent of resection or conversion of inoperable disease to operable status, was achieved in 11 out of 15 patients treated [4]. (4) Multiple primary lung cancer [5].

In Japan, PDT for early stage bronchogenic carcinoma is most common and its indications have already been determined [6]. However, in spite of the recent advances in the diagnostic techniques and establishment of the mass screening system, most lung cancers are still detected in the advanced stage. As would be expected, better therapeutic results were obtained in all resected cases of lung cancer than in nonresected cases [7]. Therefore, it is necessary to increase the number of operable cases of lung cancer to improve the survival rate. In addition, even after curative resection, 15% of patients eventually died of poor postoperative pulmonary function. In patients with limited pulmonary function, it would therefore be beneficial to reduce the extent of resection, thereby preserving the pulmonary function. In this paper, preoperative PDT will be discussed as one of the options to increase the indication of PDT.

### PATIENTS AND METHODS

### Patient Selection

An attempt to increase operability to reduce the extent of operation was made in 26 lung cancer patients ages ranging from 51 to 71 years of whom 5 patients had stage IA lung cancer, 2 with stage IB, 1 with stage IIA, 6 with stage IIB, 6 with stage IIIA, 5 with stage IIIB and 1 with stage IV lung cancer. Histologically, 22 had squamous cell carcinoma, 2 had adenocarcinoma, and 2 had large cell carcinoma (Table I). In cases of stage I, there were 4 cases of tumor invasion to the bifurcation of the right upper bronchus and trunks intermedius and 3 cases of tumor invasion of the main bronchus. In cases of stage II, 5 cases of tumor invaded to either right or left main bronchus but no invasion occurred at the site 2 cm from the carina, and 2 cases of tumor invasion of carina which were classified as stage IIB. There were 3 cases with direct tracheal invasion from the primary foci; 3 cases with endobronchial polypoid tumor or invasion of the carina; and 20 cases of polypoid tumor or invasion of the main bronchi (Table II). The invasive foci were diagnosed endoscopically as superficial mucosal invasion.

TABLE I Preoperative PDT for lung cancer

| | |
|---|---|
| No. of cases | 26 |
| Sex | Male: 24; Female: 2 |
| Age (Year) | 51–71; Ave.: 60.5 |
| Histological type | Squamous cell ca.: 22,<br>Adenoca.: 2, Large cell ca.: 2 |
| Clinical stage | IA 5 (main br: 1, 2nd carina: 4)<br>IB 2 (main br: 2)<br>IIA 1 (main br: 1)<br>IIB 6 (main br: 6)<br>IIIA 6 (main br: 5, 2nd carina: 1)<br>IIIB 5 (main br: 2, trachea: 3)<br>IV 1 (carina: 1) |

The overall survival curves for 5 patients who were classified as p-T3N0M0 (T3: main bronchus invasion) received preoperative PDT and subsequently underwent lobectomy or sleeve lobectomy and 11 patients who were classified as p-T3N0M0 (T3: main bronchus invasion) receiving surgical resection alone were compared. Each case was not randomized, however, the patients in both groups had similar background. Patients in both groups had squamous cell carcinoma and were treated within same period. Age distribution was close (averaging 60.5 years of age for the preoperative PDT group and 59.0 years for surgery alone group). The survival curves were measured by the Kaplan–Meier method.

### Procedure

Bronchoscopical PDT is performed with topical anesthesia approximately 48 h after the intravenous injection of 2.0 mg/kg body weight of Photofrin [8]. After injecting Photofrin, the patients are instructed to avoid direct sunlight for at least two weeks. The laser beam (630 nm wavelength) is transmitted via

TABLE II Preoperative PDT

| Tumor location | No. of cases | Operation method after PDT | |
|---|---|---|---|
| Trachea | 3 | Sleeve lobectomy | 1 |
| | | Pneumonectomy | 1 |
| | | Tracheoplasty | 1 |
| Carina | 3 | Sleeve lobectomy | 1 |
| | | Pneumonectomy | 1 |
| | | Exploratory thoracotomy | 1 |
| Main br. or 2nd carina | 20 | Lobectomy | 7 |
| | | Sleeve lobectomy | 10 |
| | | Pneumonectomy | 3 |
| Total | 26 | | 26 |

a quartz fiber (400 mm) inserted through the instrumentation channel of a fiberoptic bronchoscope. The fiber tip at 1–2 cm from a perpendicular target yields a circular area of illumination of 4–8 mm. The power output at the fiber tip was adjusted to 100–400 mW/cm$^2$ in cases using the argon dye laser. Using the excimer dye laser, the frequency was 30 Hz and the energy was adjusted to 4 mJ/pulse. For surface irradiation of early stage lung cancer, illumination time generally ranged from 10–40 min, giving energy densities of 100–800 J/cm$^2$. However, in advanced obstructing lung cancer, interstitial irradiation is performed with the fiber tips inserted into the tissue [9]. After PDT procedure, bronchial toilet should be performed every 2 or 3 days for 1 week. The effectiveness of PDT was evaluated both bronchoscopically and histologically 2 weeks after the PDT, and operation was performed 2–9 weeks after the PDT.

### RESULTS

Among 3 cases with tracheal invasion, sleeve lobectomy of the right upper lobe was performed in 1 case, pneumonectomy in 1 case and tracheoplasty in the remaining 1. Among 3 cases of carinal invasion by tumor, 1 underwent right upper sleeve lobectomy and 1 patient underwent left pneumonectomy, but the remaining 1 patient ended up into exploratory thoracotomy because of serious hilar lymph node involvement. In 20 cases with tumor invasion to the main bronchi, lobectomy was performed in 7 cases, and sleeve lobectomy in 10 cases, in order to preserve pulmonary function. However, pneumonectomy was performed in the remaining 3 cases because of extensive hilar lymph node involvement (Table II). Overall, the initial purpose of PDT, i.e., either reduction of extent of resection or conversion of inoperable disease to operable status, was achieved in 22 out of 26 patients treated. In 4 out of 5 patients with originally inoperable disease, conversion to an operable condition was achieved by PDT. Although 21 patients were originally candidates for pneumonectomy, it became possible to reduce the extent of resection to lobectomy or sleeve lobectomy in 18 cases (Table III). However, 3 patients subsequently died as a result of distant metastasis, and in 2 other cases, recurrence was recognized.

TABLE III Results of preoperative PDT

| | |
|---|---|
| Increase operability | |
| Inoperable to operable | 4 |
| Reduce the extent of resection | |
| Pneumonectomy to sleeve lobectomy | 11 |
| Pneumonectomy to lobectomy | 7 |
| No change | 4 |

The overall survival curves for 5 patients who received preoperative PDT and surgery, and 11 patients who received surgical resection alone were shown in Fig. 1. The survival rate of preoperative

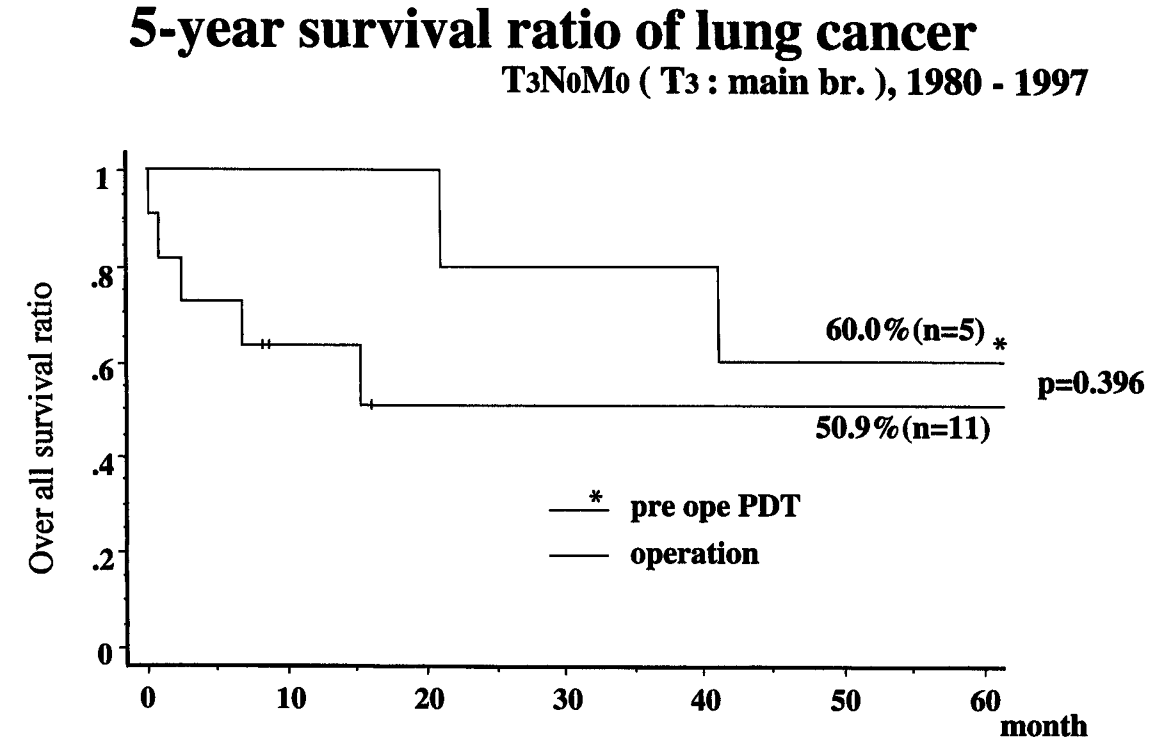

FIGURE 1 The overall survival curves for 5 patients who received preoperative PDT and surgery and 11 patients who received surgical resection alone were shown. The survival rate of preoperative PDT group and surgery alone group for 5 years was 60.0% and 50.9% respectively.

PDT group and surgery alone group for 5 years was 60.0% and 50.9% respectively.

A typical case of squamous cell carcinoma of the lung is shown in Fig. 2. The patient, a 51-year-old male, had a polypoid tumor obstructing the orifice of the right main bronchus with invasion to the lateral wall of the trachea and truncus intermedius. This tumor was initially considered inoperable and treated with PDT. Later on, the base of the tumor invasion in the lateral part of the trachea and truncus intermedius disappeared following PDT. Subsequently, upper sleeve lobectomy could be done 6 weeks after PDT.

### DISCUSSION

The past decade has seen a growing acceptance of PDT, a relatively new modality used in the treatment of cancer. This method has been used to treat a wide variety of malignancies in over 3000 patients worldwide. Approximately 90 institutions and 180 investigators throughout the world employ PDT. Five hundred patients have undergone PDT for endobronchial malignancy. There has been remarkable consistency in results of various investigator, showing CR + PR rates ranging from 70% to 100% [10]. In Japan, PDT with Photofrin and excimer dye laser obtained government approval in October 1994 and finally obtained national insurance reimbursement status in April 1996 [11].

In our basic studies with PDT, at least 1 cm tumor necrosis was noted in all the cases [12]. This observational study lead us to use PDT preparatively to reduce the extent of the surgical resection and convert inoperable cases to operable ones. In our present study, the objective of preoperative PDT was achieved in 22 out of 26 patients. The 18 cases

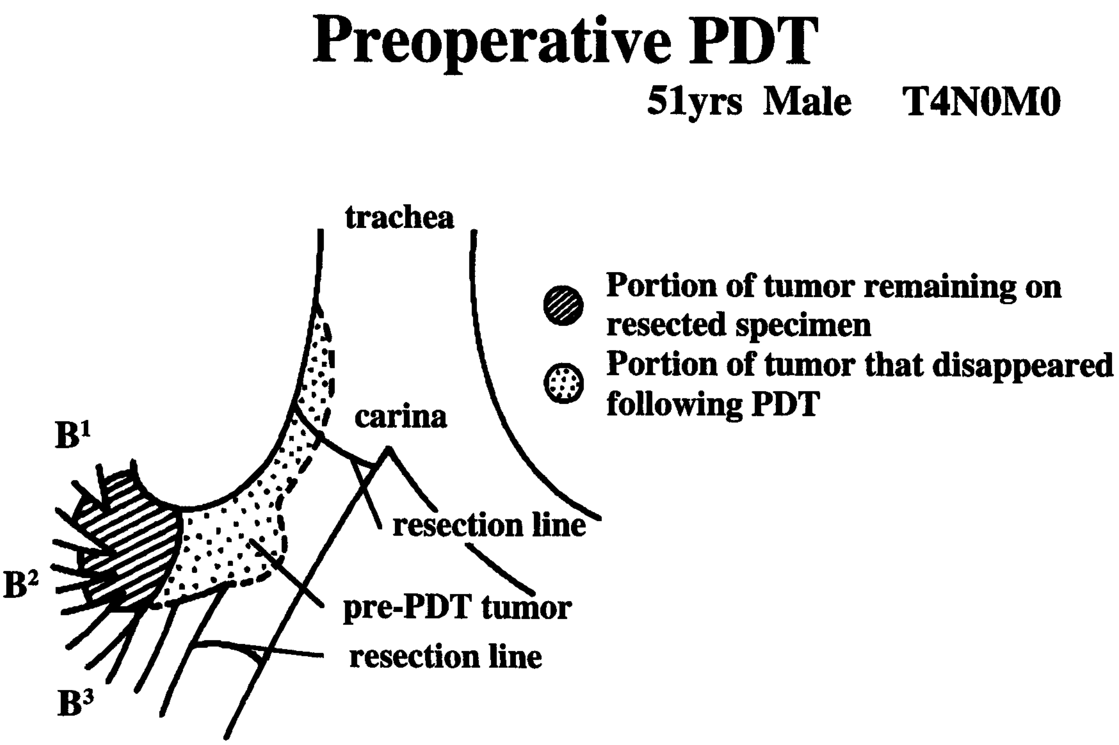

FIGURE 2 A typical case of preoperative PDT. The patient, a 51-year-old male, had a squamous cell carcinoma obstructing the orifice of the right main bronchus with invasion to the lateral wall of the trachea and truncus intermedius. Base of the tumor invasion in the lateral part of trachea and truncus intermedius disappeared following PDT. Subsequently, sleeve upper lobectomy could be done 6 weeks after PDT.

which were initially planned for pneumonectomy underwent successful lobectomy. However, 3 of them subsequently died as a result of distant metastasis, and in 2 other cases, recurrence was recognized. These results indicate the need for an accurate determination of the status of the patients before PDT. In terms of selecting patients for the procedure, endoscopic observation, brushing and biopsy are important. These examinations should confirm that the lesion is invading superficially, in which case the histological type is generally squamous cell carcinoma.

In our hospital, the T3 (main bronchus invasion) cases normally underwent surgery with a survival rate of 50.9%. In our present study, the T3 (main bronchus invasion) N0M0 cases which were planed initially for pneumonectomy underwent preoperative PDT and subsequently underwent lobectomy. The survival rate in this group of patients remarkably increased to 60.0%. Despite the fact that the number of cases in this study was limited, the result is very inspiring for further study.

Various attempts have been made to develop methods to improve the condition of lung cancer patients so that the extent of resection can be limited. These include preoperative systemic chemotherapy, bronchial arterial infusion [13] and preoperative radiotherapy [14]. Owing to promising results with the experimental and clinical applications of PDT, we applied this method for the purpose of limiting the extent of resection or for converting inoperable status to operable. In regard to this, one of the main advantages of PDT is that it is primarily tumor-specific and has negligible side effects on normal tissue.

The success from clinical trials using PDT for treatment of cancers offers encouragement for its future use. More stable, definitive and more successful results will be obtained if new dyes which distribute more equally in the tumor tissue and deeper tissue penetration by longer wavelength beams are used.

### *References*

[1] Hayata, Y., Kato, H., Konaka, C. *et al.* Hematoporphyrin derivative and laser photoradiation in the treatment of lung cancer. *Chest* 1982; **81**: 269–277.

[2] Kato, H., Okunaka, T. and Shimatani, H. Photodynamic therapy for early stage bronchogenic carcinoma. *J. Clin. Laser Med. Surg.* 1996; **14**: 235–238.

[3] Okunaka, T., Kato, H., Konaka, C. *et al.* PDT for advance bronchogenic carcinoma: PDT vs YAG laser treatment (Japanese). *Jpn. J. Bronchology* 1993; **15**: 733–738.

[4] Kato, H., Konaka, C., Ono, J. *et al.* Preoperative laser photodynamic therapy in combination with operation in lung cancer. *J. Thorac. Cardiovasc. Surg.* 1985; **90**: 420–429.

[5] Okunaka, T., Kato, H., Konaka, C. *et al.* Photodynamic therapy for multiple primary bronchogenic carcinoma. *Cancer* 1991; **68**: 253–258.

[6] Kato, H., Horai, T., Furuse, K. *et al.* Photodynamic therapy for cancers: a clinical trial of Porfimer sodium in Japan. *Jpn. J. Cancer Res.* 1993; **84**: 1209–1214.

[7] Mountain, C.F. The biologic operability of stage III non-small cell lung cancer. *Ann. Thorac. Surg.* 1985; **40**: 60–64.

[8] Kessel, D. and Cheng, M. On the preparation and properties of dihematoporphyrin ether, the tumor localizing component of HpD. *Photochem. Photobiol.* 1985; **41**: 277–282.

[9] Yamamoto, H., Kato, H., Okunaka, T. *et al.* Photodynamic therapy with the excimer dye laser in the treatment of respiratory tract malignancies. *Lasers Life Sci.* 1991; **4**: 125–133.

[10] Marcus, S.L. and Dugan, M. Global status of clinical photodynamic therapy: the registration process for a new therapy. *Lasers Surg. Med.* 1992; **12**: 318–324.

[11] Furuse, K., Fukuoka, M., Kato, H. *et al.* A prospective phase II study on photodynamic therapy with Photofrin II for centrally located early stage lung cancer. *J. Clin. Oncol.* 1993; **11**: 1852–1857.

[12] Okunaka, T., Kato, H., Konaka, C. *et al.* A comparison between argon-dye and excimer-dye laser for photodynamic effect in transplanted mouse tumor. *Jpn. J. Cancer Res.* 1992; **83**: 226–231.

[13] Neyazaki, T., Ikeda, M., Seki, Y. *et al.* Bronchial artery infusion therapy for lung cancer. *Cancer* 1969; **24**: 912–922.

[14] Scherman, D.M. and Weichselbaum, R.R. The use of preoperative radiation therapy in the treatment of lung carcinoma. *Cancer Treat. Res.* 1981; **1**: 63–73.

